# Dietary supplementation with a novel acidifier sodium diformate improves growth performance by increasing growth-related hormones levels and prevents *Salmonella enterica* serovar Pullorum infection in chickens

**DOI:** 10.3389/fvets.2024.1433514

**Published:** 2024-07-17

**Authors:** Yufan Sun, Xiaofen Zhang, Weiyao Han, Weilian Liao, Jing Huang, Yong Chen, Hengzhang Li, Xiabing Chen, Qi Huang, Rui Zhou, Lu Li

**Affiliations:** ^1^State Key Laboratory of Agricultural Microbiology, College of Veterinary Medicine, Huazhong Agricultural University, Wuhan, China; ^2^Key Laboratory of Preventive Veterinary Medicine in Hubei Province, The Cooperative Innovation Center for Sustainable Pig Production, Wuhan, China; ^3^Animal Disease Prevention and Control Center of Jiangle, Sanming, China; ^4^Veterinary Population Medicine, College of Veterinary Medicine, University of Minnesota, Saint Paul, MN, United States; ^5^Alliance Biotech Co., Ltd., Sanming, China; ^6^Institute of Animal Husbandry and Veterinary Science, Wuhan Academy of Agricultural Sciences, Wuhan, China; ^7^International Research Center for Animal Disease, Ministry of Science and Technology of the People’s Republic of China, Wuhan, China

**Keywords:** sodium diformate, chickens, growth performance, growth hormone, intestinal microbiota, *Salmonella*

## Abstract

Since the use of antibiotics as growth promoters in animal feed has been restricted or banned in several countries, finding suitable alternatives is crucial for maintaining animal health. In this study, a novel formate acidifier named sodium diformate (NaDF) was synthesized, and the effects on growth performance and the prevention effects against *Salmonella enterica* serovar Pullorum infections in chickens were assessed. In broilers, NaDF supplementation improved growth performance, as evidenced by increased body weights and reduced feed conversion ratios. At 38 days of age, NaDF supplementation increased the levels of growth-hormone and ghrelin in the serum, lowered pH values in the gut, improved duodenal morphology, as shown by increased villus length/crypt depth ratios. NaDF also modulated the abundance of beneficial and harmful bacteria without changing the general microbiota diversity and short-chain fatty acids levels, which would be beneficial for maintaining gut homeostasis during its use. NaDF exhibited a broad spectrum of antibacterial activity *in vitro*. Supplementation with NaDF effectively decreased *S*. Pullorum colonization in the cecum, liver and spleen in chickens, and mitigated pathological changes in the tissues. Therefore, as a novel acidifier, NaDF can improve chicken growth performance by increasing growth-related hormones levels while maintaining the diversity of gut microbiota, and also resist intestinal bacterial infection. These results provided evidences for the application of NaDF as an effective and safe animal feed in poultry farming.

## 1 Introduction

In recent decades, sub-therapeutic antibiotics have been used in animal feed to improve growth performance, prevent infections and reduce production costs (1). However, this practice may lead to the bacteria evolution and an increase in drug-resistant pathogens, thereby directly or indirectly contributing to the increase in human infections caused by antibiotic-resistant bacteria (2, 3). Several countries have limited or banned the use of antibiotics as feed additives in food animals (4). Sweden was the first country to ban the use of antibiotics as growth promoters in 1986, and European Union banned their use in 2006 (4). China banned the use of antibiotics as growth promoters in animal feed since 2020 (5). This could lead to an increased risk of animals contracting diseases for a period of time, as well as the re-emergence of diseases in the eradication phase. Hence, the development and application of antibiotic substitute is essential to promote animal growth and resist bacterial infections.

Over the past five decades, organic acids have received considerable attention as feed additives in animal production (6). Among these products, formic acid (FA) has been widely used to improve poultry growth performance and limit the colonization of foodborne pathogens in both poultry feed and the gastrointestinal tract (GIT) (7, 8). FA promotes nutrient utilization in poultry by enhancing digestive enzyme activities, facilitating mineral absorption and improving GIT morphology (9–11). FA also promotes the immune responses of broilers impaired by rapid growth (12, 13). Concurrently, FA exerts a direct bactericidal effect by decreasing environmental pH values, thereby creating an unfavorable environment for pH-sensitive pathogens (14, 15). Furthermore, FA has been found to modulate the composition of the intestinal microbiota and to promote the colonization of beneficial bacteria (16). However, FA is corrosive with undesirable odors, and is easily absorbed by stomach, making it hard to reach the intestine. Thus, formate acidifiers have been selected as substitutes for FA (7).

Potassium diformate (KDF, HCOOH/HCOOK; molecular weight, 130.14 g/mol) is a white crystalline compound consisting of FA and potassium formate linked via hydrogen and covalent bonds. It is highly hygroscopic and easily soluble in water, with low volatility, noncorrosive and no undesirable odor. It is dissociated into formic acid and potassium ions in the stomach after being ingested by animals and effectively addresses the problem of the inability of FA to reach the intestine (17). KDF is the first substitute approved as a non-antibiotic growth promoter by the European Union [Commission Reg (EC) number 1334/2001]. Dietary KDF effectively improves animal growth performance and nutrient utilization. In broiler, KDF supplementation improves growth performance, as well as immune and intestinal health (18). In weaning piglets, dietary KDF can increase average daily gain (ADG) and decrease feed conversion ratio (FCR) (19, 20). KDF has also been found to improve growth performance and carcass quality in growing-finishing pigs (21). In different species of fish, dietary KDF improves growth performance, by enhancing the activity of digestive enzymes and promoting nutrient absorption (22–24). On the other hand, KDF demonstrates anti-infection effects against various enteric pathogens in animals. In our previous study, it has been demonstrated that addition of KDF in feed or drinking water reduced the bacterial colonization and the degree of pathological changes after *S.* Pullorum infection in laying hens (25). In weaning piglets, KDF significantly reduced GIT bacterial loads after *Salmonella Derby* or *Escherichia coli* infection, alleviating intestinal damage and maintaining the intestinal barrier (26, 27). Similarly, in aquatic species, the mortality of *Oreochromis niloticus* fed with KDF was lower than that of the basal diet when infected with *Aeromonas hydrophila* or *Francisella noatunensis* subsp. *Orientalis* (28, 29). Based on these findings, the effectiveness and cost of FA deserves to be further optimized.

In this study, we synthesized a new type of formate acidifier named Sodium diformate (NaDF, HCOONa HCOOH; molecular weight, 114 g/mol), which is formed by the combination of FA and a modified fiber hydrogen bond carrier. Compared with KDF, NaDF showed more excellent acidification capacity, resulting in the release of greater number of hydrogen ions per kilogram. In addition, NaDF exhibited high buffering capacity and ceased to release at pH values below 3.5, thereby being expected to protect the acid-secreting ability of the stomach. Importantly, NaDF has a lower production cost. Experiments were conducted to investigate the effect of NaDF on growth performance, levels of growth-related hormones, intestinal morphologies, microbiota compositions and short-chain fatty acid (SCFA) levels of chickens. Its ability to prevent *S.* Pullorum infection of chickens in term of bacterial loads and pathological changes was also determined. The results provided evidences for the dual function of NaDF in growth promotion and anti-infection in chickens.

## 2 Materials and methods

Two studies were conducted to assess the impact of NaDF on growth performance and resistance to intestinal diseases in chickens. The first study aimed to evaluate the effect of dietary NaDF on the growth performance, intestinal health and microbiota compositions of broilers, while study 2 aimed to assess the bacteriostatic activity of NaDF *in vitro* and its preventive effect on Salmonella infection in chicks.

### 2.1 Study 1

#### 2.1.1 Detection of NaDF acidification and buffering capacity

NaDF (synthesized in this study) and KDF (a positive control) were provided by Alliance Biotech Co., Ltd. (Sanming, Fujian, China), and were all greater than 98% pure. Water used in the experiments complied with the water for analytical laboratory use and specification and test methods (GB/T 6682–2008, China), and the experiments were conducted at 25°C. Initial solutions of NaDF or KDF were obtained by dissolving 2 g of sample in 70 mL of water. To detect the acidification capacity, different volumes of 0.5057 mol/L NaOH were added to initial solutions. The pH values were determined until it suddenly changed. To detect the buffering capacity, different masses of NaDF or KDF were added to 200 mL of water and the pH values were determined.

#### 2.1.2 Experimental design and diets

A total of 672 one-day-old Arbor Acres broiler chickens with an initial average body weight (BW) of 40.48 ± 0.01 g were used and randomly divided into two groups: (i) Con group (basal diet) and (ii) NaDF group (basal diet +1 g/kg NaDF). Each group consisted of 8 replicates with 42 broilers per replicate. The male to female ratio was maintained at 1:1. All broilers were housed in pens 60 cm above the ground and covered with a plastic net. Each replicate was housed individually at a density of 14 broilers per square meter.

All broilers were kept and managed in an environmentally controlled house, ensuring appropriate temperature, humidity, light and hygiene, in accordance with the hygienic requirements for broilers. The broilers had free access to feed and water throughout the entire experimental period. The basal diets were formulated following Chinese chicken feeding standards (NY/T 33–2004) (30) (Table 1). The diets were in crumbled form for days 1–15, small pellets (2 mm diameter) for days 16–21, and large pellets (3 mm diameter) from day 22 until the end of the experiment.

**Table 1:** Composition of broiler basal diets.

Items	d 1–21	d 22–38
Ingredient, %		
Corn	49.14	42.79
Soybean meal (46% CP)	32.14	31.99
Cottonseed meal (46% CP)	2.00	2.00
Corn gluten meal (60% CP)	2.00	4.00
Wheat powder (CP 15%)	10.00	10.00
Limestone	1.20	1.22
Dicalcium phosphate	1.10	0.98
Duck oil	0.92	5.71
Sodium chloride	0.20	0.20
Premix^1^	1.30	1.11
Total	100.00	100.00
Nutrient levels^2^, %		
Dry matter	88.40	89.10
Crude protein	23.00	23.50
Calcium	0.80	0.78
Total phosphorus	0.64	0.64
Available phosphorus	0.30	0.28
Lysine	1.40	1.44
Methionine	0.60	0.58
Metabolizable energy, kcal/kg	2,850	3,100

^1^Provided per kg of diet: vitamin A acetate, 8,000 IU; vitamin D3, 2000 IU; vitamin E acetate, 10 IU; vitamin K3, 1 mg; vitamin B2, 9 mg; vitamin B6, 4 mg; vitamin B12, 0.015 mg; thiamine nitrate, 2 mg; niacin, 20 mg; D- biotin, 0.09 mg; D- calcium pantothenate, 9 mg; folic acid, 0.95 mg; Cu, 8 mg; Fe, 80 mg; Mn, 60 mg; Zn, 60 mg; I, 0.5 mg; Se, 0.3 mg. ^2^Calculated values based on the analyzed data of experimental diets.

#### 2.1.3 Growth performance measurements and sample collections

BW and feed intake were recorded weekly for each replicate of broilers to calculate ADG, average daily feed intake (ADFI) and FCR on a per cage basis.

On day 38, one broiler from each replicate was randomly selected for euthanasia (n = 8). Blood samples were collected and centrifuged at 3,000 × *g* for 15 min at 4°C and the serum stored at −80°C for subsequent analysis of hormone levels. Duodenal and cecum tissues were collected and fixed in 4% paraformaldehyde for observations and measurements of intestinal histomorphology. The contents of the duodenum and cecum were collected under aseptic conditions, immediately snap-frozen in liquid nitrogen and then stored at −80°C for the analysis of intestinal microbiota and SCFAs levels. Additionally, the contents of the proventriculus, gizzard, duodenum, jejunum, ileum, cecum and rectum were collected, and the pH values of each section were determined using a digital pH meter.

#### 2.1.4 Detection of growth hormone and ghrelin levels

Serum GH and ghrelin levels were assayed using commercial ELISA kits (Jiangsu Meimian Industrial Co., Ltd., Jiangsu, China). Microtiter plate wells were coated with purified chicken GH and ghrelin antibodies. Standards and samples were added to coated wells, and the samples were diluted fivefold. Antibodies are combined with HRP labels to form antibody–antigen–enzyme-antibody complexes, and TMB substrate solution was added to develop the color. Absorbance at 450 nm was measured after colorization using a microplate reader. The serum hormone levels were determined from the standard curve by converting the corresponding absorbance.

#### 2.1.5 Intestinal histomorphology observations

Duodenal and cecum samples were fixed with paraformaldehyde and dehydrated in alcohol, then embedded in paraffin and sectioned into 4 μm sections. Sections were then stained with hematoxylin and eosin, and morphological changes were observed under a light microscope (Nikon Eclipse CI). Six sections of each group and six villi of each section were randomly selected. The villus length (VL) and crypt depth (CD) were measured using Image-pro plus 6.0 (Media Cybernetics, Inc., Rockville, MD, United States), and the villus length to crypt depth ratio (VL/CD) was also calculated.

#### 2.1.6 DNA extraction, 16S rRNA gene sequencing and bioinformatics analysis

Total genomic DNA from duodenal and cecal contents of broilers was extracted using a TIANamp stool DNA kit (TIAN GEN). The quality and concentration of DNA were detected by 1.0% agarose gel electrophoresis and a NanoDrop® ND-2000 spectrophotometer (Thermo Scientific Inc., United States). The hypervariable region V3-V4 of the bacterial 16S rRNA gene was amplified with primer pairs 338F (5’-ACTCCTACGGGAGGCAGCAG-3′) and 806R (5’-GGACTACHVGGGTWTCTAAT-3′) with an ABI GeneAmp® 9,700 PCR thermocycler (ABI, CA, United States). The PCR product was purified using the AxyPrep DNA Gel Extraction Kit (Axygen Biosciences, Union City, CA, United States) and quantified using a Quantus™ Fluorometer (Promega, USA). Purified amplicons were pooled in equimolar amounts and paired-end sequenced on an Illumina NovaSeq PE300 platform (Illumina, San Diego, United States) according to standard protocols by Majorbio Bio-Pharm Technology Co. Ltd. (Shanghai, China).

The sequences were quality-filtered by fastp version 0.19.6 and merged by FLASH version 1.2.7. The optimized sequences were then clustered into operational taxonomic units (OTUs) using UPARSE 7.1 with a 97% sequence similarity level. The taxonomy of each OTU representative sequence was analyzed by RDP Classifier version 2.11 against the 16S rRNA gene database^1^ using confidence threshold of 0.7. Bioinformatic analysis of the intestinal microbiota was carried out using the Majorbio Cloud platform.^2^ Alpha diversity (Chao and Shannon index) was calculated with Mothur v1.30.2 to analyze the microbial diversity and richness, and the Wilcoxon rank sum test was used to test for differences between the two groups. Beta diversity was determined to analyze the similarity among the microbial communities using principal coordinate analysis (PCoA) and nonmetric multidimensional scaling analysis (NMDS) based on Bray–Curtis distance (Vegan v2.5–3 package). Analysis of similarities (ANOSIM) and Adonis analysis were used to assess statistical significance (Vegan v2.5–3 package). Linear discriminant analysis (LDA) (LDA score > 2.0, *p* < 0.05) and the Wilcoxon rank sum test were used to analyze the differences in bacterial abundances among groups.^3^

#### 2.1.7 Determination of SCFAs levels in the duodenum and cecum contents

Intestinal content samples (25 mg) were accurately weighed into 2 mL grinding tubes, and 800 μL of 0.5% phosphoric acid water containing the internal standard 2-ethylbutyric acid (10 μg/mL) was added. Samples were frozen and ground at 50 Hz for 3 min, which was repeated twice, followed by ultrasonication for 10 min and centrifugation at 4°C and 13,000 × *g* for 15 min. Then, 200 μL of the supernatant aqueous solution was transferred into a 1.5 mL centrifuge tube, and 0.2 mL of N-butanol solvent was added for extraction. Lastly, samples were vortexed for 10 s and ultrasonicated on ice for 10 min, followed by centrifugation at 4°C and 13,000 × *g* for 5 min. Supernatants were then carefully transferred to sample vials for analysis. The analysis was performed using an Agilent 8890B gas chromatograph coupled to an Agilent 7000D mass selective detector at Majorbio Bio-Pharm Technology Co. Ltd.

### 2.2 Study 2

#### 2.2.1 Minimum inhibitory concentration assays

The MIC of NaDF against common pathogenic bacteria in the farm was detected *in vitro*. The bacterial strains were stored in our laboratory. The microbroth dilution method was employed to determine the MIC (14).

#### 2.2.2 Experimental design

Chickens were hatched from SPF White Leghorn chicken eggs (Boehringer Ingelheim Vital Biotechnology Co, Ltd. Beijing, China) at 37°C in an incubator for this study. NaDF was provided by Alliance Biotech Co., Ltd. (Sanming, Fujian, China) and was greater than 98% pure. All chickens were raised in SPF chicken isolators and provided feed and water *ad libitum*. A total of 72 1-day-old SPF Leghorn chickens were randomly assigned into four groups (*n =* 18): (i) NC group (basal diet and water without infection), (ii) SP group (basal diet and water with *S.* Pullorum infection), (iii) NaDF-diet (SP) group (basal diet +5 g/kg NaDF with *S.* Pullorum infection) and (iv) NaDF-water (SP) group (1 g/L NaDF in water with *S.* Pullorum infection).

#### 2.2.3 *Salmonella* infection assay

*S.* Pullorum C79-13 was obtained from the China Institute of Veterinary Drug Control. The strain was cultured aerobically in tryptic soy broth or tryptic soy agar at 37°C. Two days after NaDF pretreatment, chickens in the infected groups were orally inoculated with 0.5 mL of *S.* Pullorum C79-13 at a dose of 1.2 × 10 ^9^ CFU per chicken, while the NC group received the same amount of phosphate-buffered saline. The chickens were fasted for 4 h before infection. Their BW, tissue bacterial loads and pathological changes were recorded on different days’ post-infection (dpi).

#### 2.2.4 Weight measurements and sample collections

The BW of chickens in all groups was measured and recorded daily until the end of the experiment. At 1.5, 3 and 6 dpi, six chickens were randomly selected and euthanized from all groups. The liver, spleen and one of the ceca were collected under aseptic conditions to determine the bacterial loads in the tissues using plate counts. In addition, the liver and another cecum were collected and fixed in 4% paraformaldehyde at 6 dpi for histopathological analysis. The methods were the same as described above in study 1.

### 2.3 Statistical analysis

Two-way ANOVA was used to do the statistical analysis of growth performance in study 1 and body weight ratio (BWR, body weight compared to the day before infection) in study 2. Two-tailed Student’s *t* tests were used to do statistical analysis between two groups unless otherwise indicated (GraphPad Prism 9.5.0, GraphPad Software, La Jolla, CA, United States). The results expressed as the means ± standard deviations (SD). ^*^ *p* < 0.05, ^**^ *p* < 0.01, ^***^ *p* < 0.001, ^****^ *p* < 0.0001.

## 3 Results

### 3.1 Study 1

#### 3.1.1 NaDF provides strong acidification and buffering capacity

The pH value of NaDF solution was significantly lower than that of KDF solution with the addition of 30 mL of 0.5057 mol/L NaOH. The pH value of the NaDF solution was suddenly increased to 8.47 by adding 35.82 mL of 0.5057 mol/L NaOH. The same change happened in KDF solution with the addition of 31.67 mL of 0.5057 mol/L NaOH. The results indicate that NaDF has a significantly higher capacity to release hydrogen ions than KDF (Supplementary Table S1). The pH value of NaDF solution stabilized at 3.50 and for KDF solution at 3.85 with increasing masses of NaDF or KDF (Supplementary Table S2).

#### 3.1.2 NaDF improves the growth performance and increases levels of growth-related hormones of broilers

The effects of NaDF on the growth performance of broilers are shown in Table 2. At 38 days of age, broilers in the NaDF group exhibited a significant increase in BW compared to the Con group (*p* < 0.05). Throughout the 1–38-day period, FCR decreased significantly in the NaDF group (*p* < 0.01). In addition, ADG tended to increase at the whole growing period (1–38 d), and FCR tended to decrease at the finishing stage (22–38 d) in the NaDF group (0.05 < *p* < 0.1). Weekly growth performance data are shown in Supplementary Table S3. On days 22–28, ADFI of broilers in the NaDF group was significantly higher than that in the Con group (Supplementary Table S3; *p* = 0.05), and ADFI in the NaDF group tended to increase at each stage of broilers growth.

**Table 2:** Growth performance of broilers fed with 1 g/kg NaDF.

Items	Con	NaDF	*p* value
BW, g			
d 21	798.55 ± 25.23	813.14 ± 23.45	0.2509
d 38	2293.80 ± 45.69^a^	2342.98 ± 45.42^b^	^*^0.0487
ADG, g/d per bird			
d 1–21	35.98 ± 1.26	36.60 ± 1.26	0.3430
d 22–38	101.16 ± 2.86	103.22 ± 2.87	0.1731
d 1–38	58.12 ± 1.89	59.67 ± 1.59	0.0976
ADFI, g/d per bird			
d 1–21	48.18 ± 1.39	48.92 ± 1.29	0.2938
d 22–38	140.37 ± 4.01	141.60 ± 2.50	0.1069
d 1–38	88.91 ± 2.78	89.94 ± 2.04	0.4121
F/G (g/g)			
d 1–21	1.34 ± 0.02	1.34 ± 0.02	0.7884
d 22–38	1.39 ± 0.01	1.37 ± 0.02	0.0792
d 1–38	1.53 ± 0.01^a^	1.51 ± 0.01^b^	^**^0.0011

Different superscripts in the same row showed significant difference. Con group (basal diets); NaDF group (NaDF in diet). BW, Body Weight; ADG, average daily gain; ADFI, average daily feed intake; F/G, feed conversion ratio. ^*^p < 0.05, ^**^p < 0.01.

**Figure 1:**
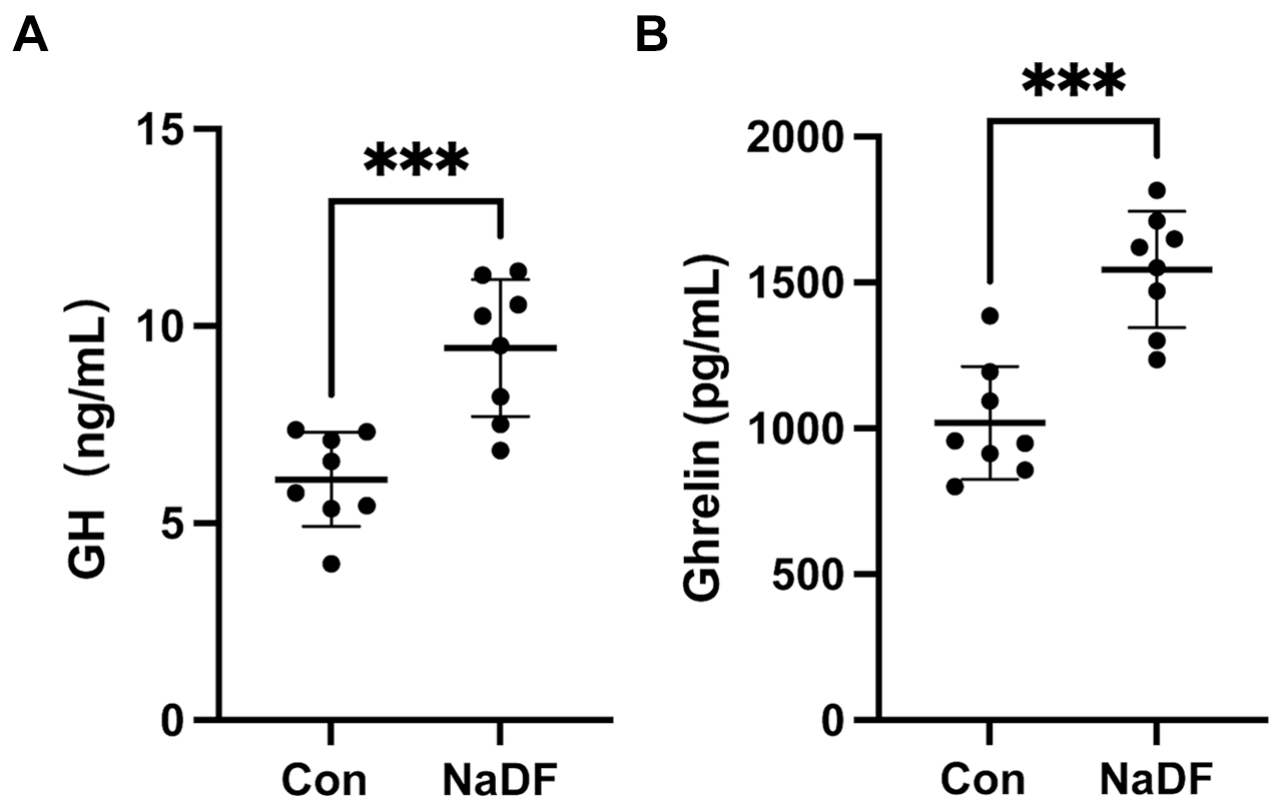
The addition of NaDF to the diet increased growth-related hormone levels of chickens in Con group (basal diets) and NaDF group (NaDF in diet). **(A)** Growth hormone levels. **(B)** Ghrelin levels. ^***^ *p* < 0.001.

Levels of growth-related hormones were also analyzed. At 38 days of age, serum GH and ghrelin levels of broilers in the NaDF group were significantly higher than those in the Con group (*p* < 0.001, Figure 1).

#### 3.1.3 NaDF reduces gastrointestinal pH values

At 38 days of age, broilers were euthanized and the pH values of different sections of the GIT were measured. Compared to the Con group, the NaDF group displayed significantly lower pH values in the GIT, with significant differences observed in the duodenum and ileum (Tables 3, *p* < 0.05).

**Table 3:** The pH values in different GIT segments of broilers.

GIT segment	Proventriculus	Gizzard	Duodenum	Jejunum	Ileum	Cecum	Rectum
Con	4.29 ± 0.30	4.32 ± 0.26	6.18 ± 0.28^a^	5.84 ± 0.31	6.89 ± 0.67^a^	6.29 ± 0.41	6.16 ± 0.47
NaDF	4.28 ± 0.57	4.26 ± 0.48	5.85 ± 0.31^b^	5.67 ± 0.61	6.19 ± 0.62^b^	6.28 ± 0.25	5.88 ± 0.43
*P* value	0.9527	0.7888	^*^0.0444	0.4936	^*^0.0469	0.9653	0.2297

Different superscripts in the same column showed significant difference, the same as below. Con group (basal diets); NaDF group (NaDF in diet). ^*^p < 0.05.

#### 3.1.4 NaDF influences intestinal histomorphology

**Figure 2:**
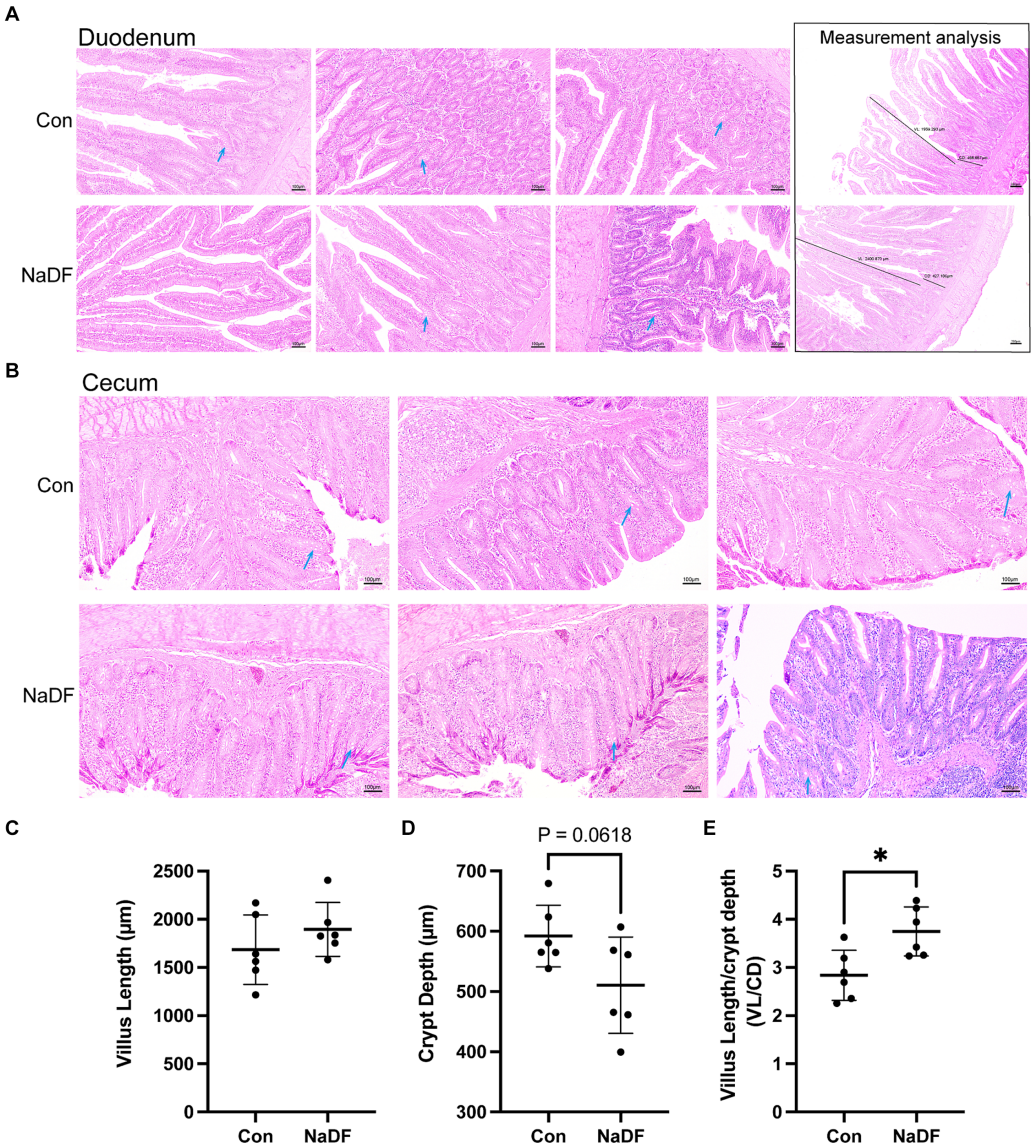
The addition of NaDF to the diet improved gut morphology. **(A)** Histomorphology and measurement analysis of the duodenum. The dark lines in the pictures on the right indicate villus length (VL) and crypt depth (CD) for measurements. **(B)** Histomorphology of the cecum. Blue arrows: goblet cells. **(C)** The villus length, **(D)** crypt depth and **(E)** VL/CD ratio of the duodenum. Con group (basal diets), NaDF group (NaDF in diet). ^*^ *p* < 0.05.

Supplementation with NaDF did not induce pathological changes in the duodenum and cecum, as observed through histomorphology analysis (Figures 2A,B). In the duodenum, there was a tendency for lower duodenal CD in the NaDF group (0.05 < *p* < 0.1). A significant increase in the ratio of VL/CD was observed in the NaDF group (*p* < 0.05, Figures 2C–E).

#### 3.1.5 The microbial diversity, abundance and levels of SCFAs in the duodenal and cecal influenced by NaDF

**Figure 3:**
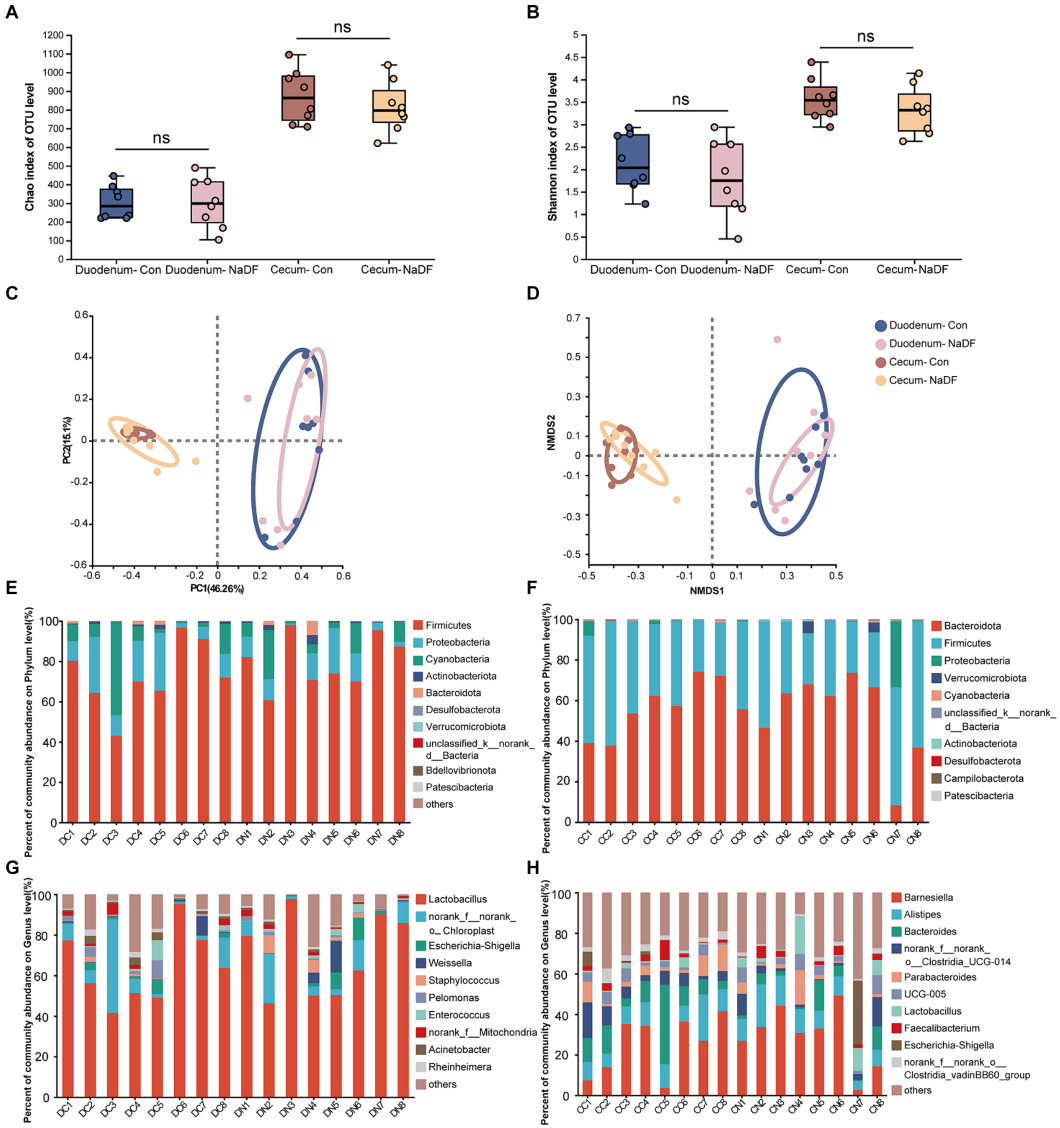
Microbial diversities of the duodenum and cecum. **(A)** Chao1 index. **(B)** Shannon index. **(C)** Principal coordinate analysis. **(D)** Nonmetric multidimensional scaling analysis. **(E-H)** Relative abundance distributions of the top 10 intestinal microbiota at the phylum and genus levels in the duodenum and cecum. DC (duodenal contents of Con group); DN (duodenal contents of NaDF group); CC (cecal contents of Con group); CD (cecal contents of NaDF group).

The α-diversity analysis, Chao1 and Shannon indices, showed no significant differences in microbial diversity between the NaDF and Con groups in both the duodenum and cecum (Figures 3A,B). Then, the similarities and differences in microbial community structure in different samples were investigated. PCoA and NMDS analysis based on Bray–Curtis distance showed no changes in duodenal and cecal microbial community structure with the addition of NaDF (Figures 3C,D). The ANOSIM and Adonis based on Bray–Curtis distance at the OTU level confirms the same result. Therefore, the relationship in gut microbiota between different groups showed no significant difference in β-diversity in either the duodenum or cecum.

The top 10 microbes at the phylum and genus levels in the duodenum and cecum are shown in Figures 3E–H. The most abundant phyla observed in the duodenum were Firmicutes (72.90%), Proteobacteria (14.59%) and Cyanobacteria (10.99%), while Bacteroidota (56.52%), Firmicutes (41.41%) and Proteobacteria (1.24%) were the dominant phyla in the cecum. At the genus level, Lactobacillus (64.04%) and norank_f_norank_o_Chloroplast (10.98%) were the most abundant in the duodenum, whereas Barnesiella (24.94%), Bacteroides (12.58%), Alistipes (11.19%), norank_f_norank_o_Clostridia_UCG-014 (6.92%), Parabacteroides (5.61%), UCG-005 (3.63%), norank_f_norank_o_Clostridia_vadinBB60_group (3.19%) and Faecalibacterium (2.88%) were the predominant genera in the cecum.

**Figure 4:**
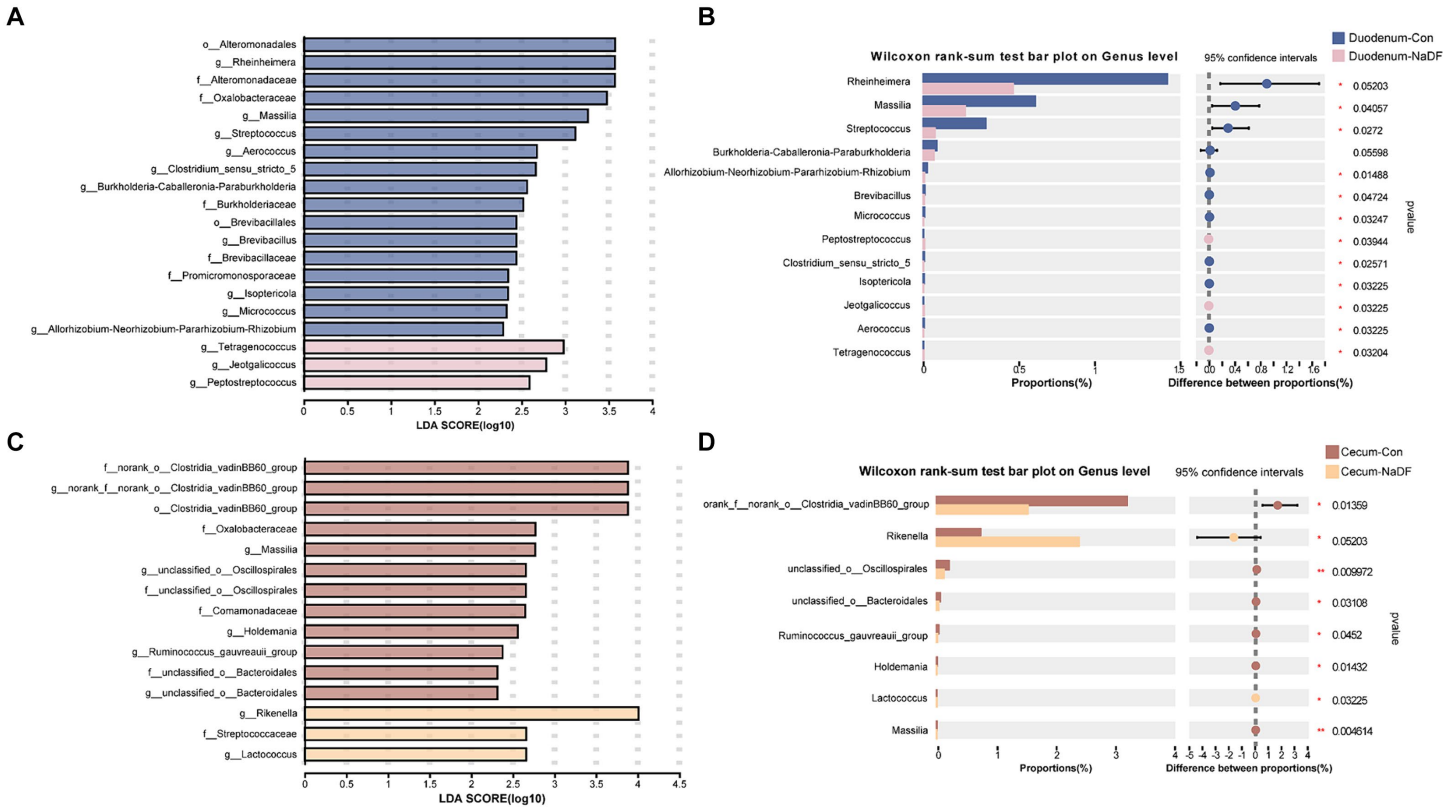
Species differences in the intestinal microbiota of the duodenum and cecum. **(A,B)** LEfSe analysis. **(C,D)** Changes at the genus level. ^*^ *p* < 0.05, ^**^ *p* < 0.01.

Although NaDF did not significantly change the microbiota diversity in the duodenal and cecal, the relative abundance of some communities was influenced by NaDF supplementation at the genus level. Linear discriminant analysis Effect Size (LEfSe) analysis identified significant bacterial taxa associated with NaDF (LDA score > 2, Figures 4A,B). Further analysis at the genus level showed that the relative abundances of Rheinheimera, Massilia, Streptococcus, Allorhizobium-Neorhizobium-Pararhizobium-Rhizobium, Brevibacillus, Micrococcus, Clostridium_sensu_stricto_5, Isoptericola and Aerococcus were significantly lower in the duodenum of the NaDF group compared to the Con group, whereas Peptostreptococcus, Jeotgalicoccus and Tetragenococcus were significantly higher (*p* < 0.05, Figure 4C). In the cecum, the relative abundances of norank_f_norank_o_Clostridia_vadinBB60_group, unclassified_o_Oscillospirales, unclassified_o_Bacteroidales, Ruminococcus_gauvreauii_group, Holdemania and Massilia were significantly decreased in the NaDF group, whereas Rikenella and Lactococcus were significantly increased in the NaDF group (*p* < 0.05, Figure 4D). The raw sequencing reads were deposited into the NCBI Sequence Read Archive (SRA) database (accession number: SRP472608).

The correlations between differential microbiota at genus level with serum growth-related hormone levels were analyzed using Spearman’s correlation analysis (Supplementary Figure S1). The level of GH was negatively correlated with the relative abundance of Micrococcus and Holdemania (*p* < 0.05). The level of ghrelin was positively correlated with the relative abundance of Tetragenococcus and Jeotgalicoccus and negatively correlated with Aerococcus and Micrococcus (*p* < 0.05).

Additionally, supplementation of NaDF in the diet caused no significant changes in the levels of major SCFAs in the broiler intestine (Supplementary Table S4).

### 3.2 Study 2

#### 3.2.1 Mics of NaDF against different pathogenic bacteria *in vitro*

The MIC values of NaDF against fifteen different pathogenic bacteria ranged from 6.2500–1.5625 mg/mL (Supplementary Table S5). The lowest MIC values were observed in *P. multocida* HB03 and *S. suis* SC19, with 1.5625 mg/mL. The highest MIC value was observed in *S. aureus* ATCC212193 with 6.2500 mg/mL. The MIC values of NaDF against Salmonella, *E. coli*, *A. pleuropneumoniae*, *C. perfringens*, *P. multocida* 9261 and *S. aureus* 1213M4A were 3.1250 mg/mL.

#### 3.2.2 Body weight losses in chickens infected with *Salmonella* were alleviated by NaDF

No death occurred after infection of *S.* Pullorum. During days 4 to 9, the BWR of chickens in the SP group was significantly lower than that in the NC group (*p* < 0.05; Table 4). While compared to the SP group, on days 3, 4, 6, 7, 8 and 9, the NaDF-treated group had a significant higher BWR (*p* < 0.05; Table 4).

**Table 4:** Changes in BWR of chickens after *S. pullorum* infection.

Group/Days	1	2	3 (Infected)	4	5	6	7	8	9
NC	1.00 ± 0.04	1.06 ± 0.05	1.07 ± 0.09^a^	1.27 ± 0.11^bc^	1.40 ± 0.10^a^	1.57 ± 0.10^a^	1.63 ± 0.10^a^	1.75 ± 0.06^a^	1.96 ± 0.07^a^
NaDF- diet (SP)	1.00 ± 0.03	1.10 ± 0.04	1.18 ± 0.04^b^	1.27 ± 0.06^c^	1.34 ± 0.05^ac^	1.42 ± 0.07^b^	1.60 ± 0.04^a^	1.78 ± 0.04^a^	1.95 ± 0.03^a^
NaDF- water (SP)	1.00 ± 0.03	1.10 ± 0.02	1.18 ± 0.03^b^	1.19 ± 0.08^b^	1.28 ± 0.11^bc^	1.35 ± 0.19^b^	1.58 ± 0.10^a^	1.72 ± 0.15^a^	1.85 ± 0.11^a^
SP	1.00 ± 0.04	1.04 ± 0.04	1.10 ± 0.05^a^	1.09 ± 0.09^a^	1.20 ± 0.08^b^	1.26 ± 0.11^c^	1.37 ± 0.06^b^	1.59 ± 0.15^b^	1.70 ± 0.07^b^

NC (basic diets); SP (basic diets, infected with S. Pullorum); NaDF- diet (SP) (NaDF in diet, infected with S. Pullorum); NaDF- water (SP) (NaDF in water, infected with S. Pullorum). ^*^P < 0.05.

#### 3.2.3 Colonization of bacteria and pathological damages in the cecum and liver of chickens infected with Salmonella were reduced by NaDF

**Figure 5:**
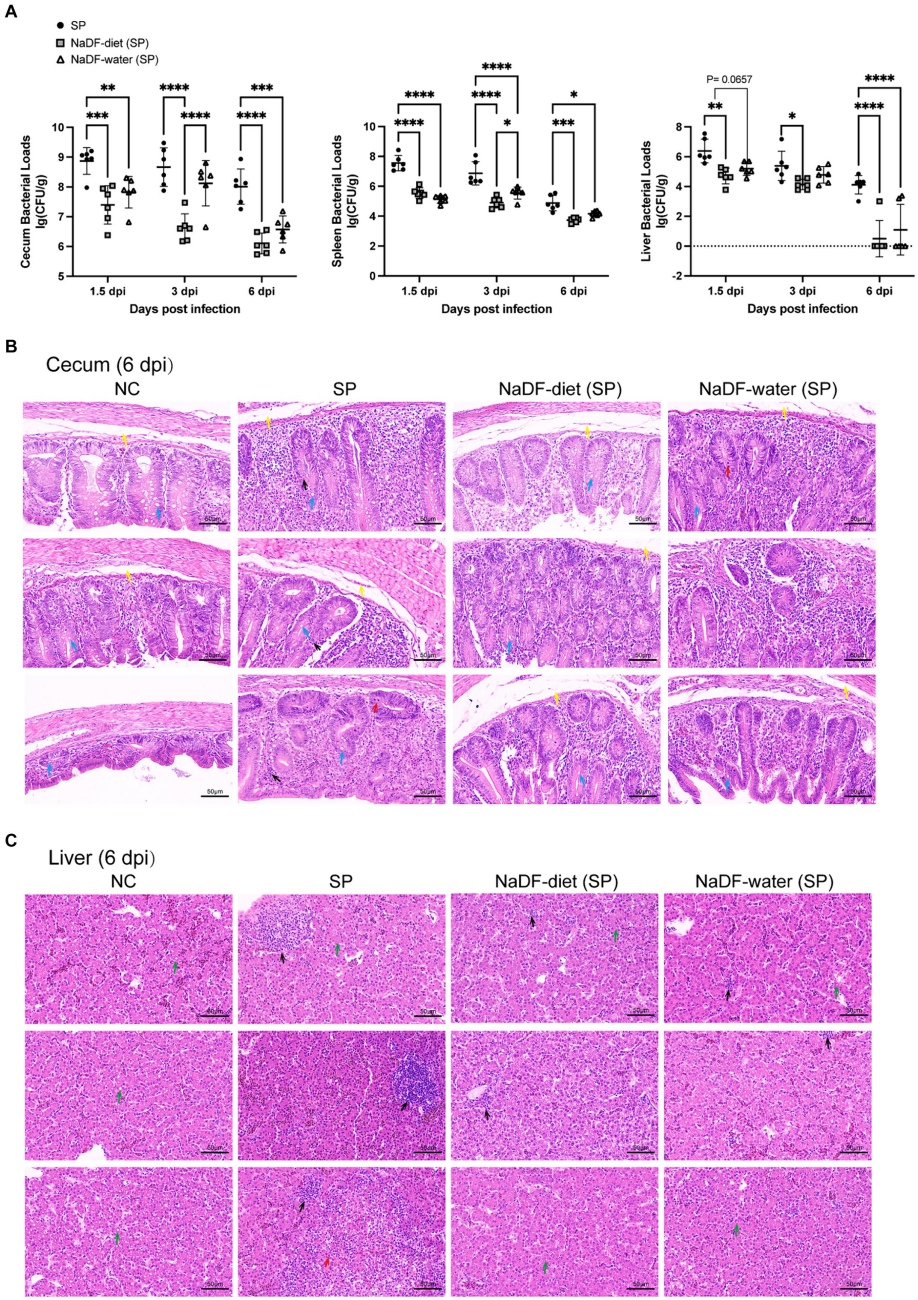
The addition of NaDF enhanced the resistance of chickens to *S.* Pullorum infection. **(A)** *S.* Pullorum loads in the cecum, spleen and liver. **(B)** Histopathological analysis of cecum and liver tissues of chickens in different groups at 6 dpi. Black arrows: inflammatory cell infiltration. Red arrows: mucosal epithelial cell necrosis and nuclear pyknosis, dissolution and disappearance. Yellow arrows: mucosal edema. Blue arrows: goblet cells. Green arrows: mild fatty degeneration with some lipid droplets in the cytoplasm. NC (basic diets); SP (basic diets, infected with *S*. Pullorum); NaDF- diet (SP) (NaDF in diet, infected with *S*. Pullorum); NaDF- water (SP) (NaDF in water, infected with *S*. Pullorum). ^*^p < 0.05; ^**^p < 0.01; ^***^p < 0.001; ^****^p < 0.0001.

The bacterial loads in tissues of chickens infected with *S*. Pullorum were analyzed on 1.5, 3 and 6dpi. Throughout these periods, the NaDF-treated group exhibited reduced *S.* Pullorum loads in the cecum, liver and spleen (Figure 5A). At the same time, the inhibition effect of supplementation of NaDF in the diet was more effective than that in drinking water (Figure 5A).

The cecum and liver tissues of chickens in the NC group appeared normal, while those in the SP group displayed evident pathological changes, including disrupted normal structure, marked inflammatory cell infiltration, cell necrosis and submucosal edema in the cecum (Figures 5C,D). However, the addition of NaDF to the feed and drinking water alleviated tissue lesions (Figures 5C,D). Following NaDF treatment, the cecum of chickens exhibited nearly normal structural integrity with minimal inflammatory cell infiltration and reduced goblet cells. Moreover, inflammatory cell infiltration in the liver was reduced.

## 4 Discussion

**Figure 6:**
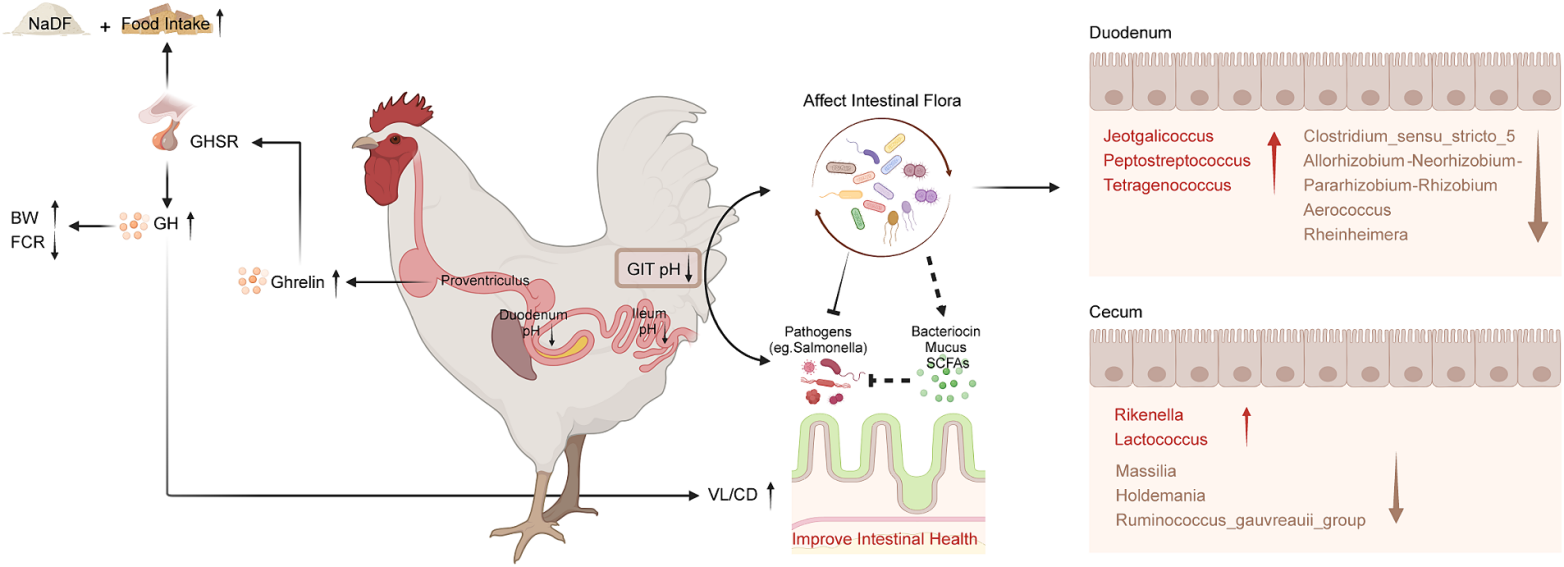
Schematic diagram of the effects of NaDF supplementation to improve growth performance and prevent Salmonella infection in chickens. BW (body weight); FCR (feed conversion ratio); GHSR (growth hormone secretagogue receptor); GH (growth hormone); GIT (gastrointestinal tract); VL (villus length); CD (crypt depth); SCFA (short-chain fatty acid).

With the policies of removal of antibiotic growth promoters from food animal production in several countries, sustainable alternatives are urgently needed to promote growth and prevent disease in food animal production. In this study, we investigated the effects of NaDF, a novel organic acid feed additive, on growth promotion in broilers and on Salmonella resistance in chicks (Figure 6). Our findings revealed that NaDF significantly increased the BW and decreased the FCR in broilers, which aligns with previous research on the growth-promoting effects of organic acids in poultry and swine (9, 17, 31, 32). In particular, the most significant effect of NaDF in reducing FCR was observed during two specific stages of broiler development, including 1–7 days and 22–28 days of age. In the first week, this effect may be due to the immaturity of the GIT of chicken, with insufficient gastric acid secretion and an unstable gut microbial community, making exogenous acid supplementation more effective (33). At 22 days of age, the diet of broilers was changed from small pellets to large pellets. The physical form of the feed influences the secretion of gastric acid and pepsin to further affect nutrient digestion (34). NaDF supplementation during this period may facilitate the digestive enzyme activity.

The primarily function of organic acids is to create lower pH environments. The lower pH in the GIT maintains the activity of pepsin and trypsin, which enhances protein utilization and nutrient digestibility (35). This may be one reason for the higher BW of broilers in the NaDF group compared to the Con group at 38 days of age. In addition, organic acids are known to maintain the integrity of the intestinal barrier through their diffusion into bacterial cells, where they dissociate and release H^+^ ions, and limiting the growth of bacteria that are less tolerant to acidic pH while promoting the growth of acid-tolerant probiotics (36). We observed that NaDF significantly reduced the pH values of the duodenum and ileum in broilers in study 1. In study 2, NaDF exhibited antimicrobial activity *in vitro* and effectively prevented Salmonella infections in chicks by reducing colonization and alleviating pathological changes of chicken tissues. These findings are consistent with our previous research that KDF can successfully prevent Salmonella infections in chickens by acidifying the GIT environment (25). Therefore, we suggest that the main reasons for the effectiveness of NaDF in preventing infections is that acidification of the GIT environment, thereby providing a direct bactericidal effect. Meanwhile, the acidification effect of organic acids is commonly thought to be more effective in the upper GIT (37), which also could be observed with NaDF supplementation in the diet. NaDF had no significant effect on the pH values of the broiler proventriculus and gizzard. This is probably attributed to the high buffering capacity of NaDF, which protects the acid-secreting ability of the stomach at low pH values. NaDF significantly reduced the pH values in the duodenum and ileum because the original pH values of these two sections are relatively higher than that in stomach. Then, with dissociation and reduced concentrations of NaDF in the lower intestine, it was not sufficient to cause a significant difference in the pH values of the cecum and rectum, compared to the Con group.

Our results demonstrated the beneficial effect of NaDF on intestinal morphology in terms of increased duodenal VL and VL/CD ratio and decreased CD. Intestinal morphology serves as an essential indicator for evaluating the digestive and absorptive function of the intestine (38). Longer small intestinal villi facilitate the absorption of nutrients such as monosaccharides, amino acids and fatty acids, and shallower crypts indicates that the intestinal epithelial cells have a higher degree of maturity and better nutrient absorption (39). Previous studies have also demonstrated that dietary supplementation with organic acids increases VL in the duodenum, jejunum and ileum and improves BW in broilers (40–42). GH can regulate the structure of intestinal villi (43). A previous study has confirmed that the villous volumes and surface areas were reduced in GH-deficient rats, which could be restored to the levels of the control rats after intraperitoneal GH administration (44). Therefore, GH promotions found in the NaDF group can account for the growth-promoting effects of NaDF in broilers.

According to the results, dietary NaDF significantly elevated the serum levels of GH and ghrelin in broilers, which has not been reported as an effect of FA supplementation previously. Central nervous system is crucial in regulating appetite, energy balance and BW (45). GH, mainly produced from the pituitary gland, plays a crucial role in regulating avian growth rate by influencing different physiological processes such as protein synthesis, cell proliferation and metabolism in muscle and adipose tissue (46, 47). Ghrelin, a peptide hormone produced by chicken proventriculus, acts as a potent stimulator of GH release and serves a hunger signal role that enhances food intake and weight gain to regulate energy balance (48–51). It has been shown that broilers with higher ghrelin levels exhibit higher plasma GH levels and BW (52). Therefore, we inferred that NaDF promotes the secretion of endogenous ghrelin, which in turn stimulates appetite and triggers the release of GH from the pituitary, thus improving the growth performance of broilers (Figure 5), which may serve as another mechanism by which NaDF promote animal growth performance.

The commensal intestinal microbiota plays important roles in the maintenance of the normal physiology of animals. Diet can affect host health by modulating GIT microbial communities (53). Our study revealed that supplementation with 1 g/kg NaDF in the diet had no significant effect on microbial alpha and beta diversity. Subsequently, we analyzed the changes in relative abundance of bacteria at the genus level. In the duodenum, the relative abundances of Jeotgalicoccus, Peptostreptococcus and Tetragenococcus were significantly higher in the NaDF group than that in the Con group. However, the relative abundances of Clostridium_sensu_stricto_5, Allorhizobium-Neorhizobium-Pararhizobium-Rhizobium, Aerococcus and Rheinheimera were significantly lower. In the genera with increased abundance, Peptostreptococcus has been shown to possess probiotic characteristics. *P. russellii* has been reported to inhibit BW decline and inflammation caused by dextran sodium sulfate (54). This bacterium can enhance the mucus barrier and utilize mucin for colonization, and produces tryptophan metabolite indole acrylic acid to maintain intestinal epithelial barrier and mitigate inflammatory responses (54). Jeotgalicoccus has been found to be positively correlated with immune function in broilers, and its relative abundance increases in the ileum of litter floor broilers with stronger immunity (55, 56). Tetragenococcus is a lactic acid- and bacteriocin-producing bacterium (57). As a probiotic, *T. halophilus* can alleviate intestinal inflammation in mice by altering gut microbiota and regulating dendritic cell activation via CD83 (58). Long-term administration of *T. halophilus* over generations has been found to promote immune activation and tolerance and enhance immunological robustness (59). In genera with decreased abundance, Clostridium_sensu_stricto_5 potentially associated with necrotic enteritis (NE), an enterotoxemic disease in poultry caused by *Clostridium perfringens*. The ban on the addition of antibiotics to feed in locations such as the European Union and China has led to an increase in NE incidence (60). Acute cases of NE have a mortality rate of up to 50% (61). Chronic NE significantly reduces the growth performance of chickens, causing intestinal ulceration and erosion, leading to serious economic losses in poultry farming (61). Allorhizobium-Neorhizobium-Pararhizobium-Rhizobium are considered to be harmful bacteria in chickens, causing intestinal inflammation (62). Aerococcus are strongly associated with infections in humans and animals. Their members cause urinary tract infections, infective endocarditis and arthritis in humans (63, 64). *A. viridans* causes pneumonia, meningitis, arthritis and urinary tract infection in pigs (65, 66) and subclinical intramammary infections in dairy cows (67, 68). But the impact of Aerococcus on poultry has not been reported. *Rheinheimera texasensis* is known to be a human pathogen bacterium that carries multiple antibiotic resistance genes (69).

The absorption and metabolism of organic acids primarily take place in the upper GIT segments of poultry and weakly affect the distal intestine, as demonstrated in the present study and our previous studies (25, 70). Hence, it is reasonable that in the cecum, the changes in the abundances of bacterial genera were less pronounced than those seen in the duodenum. We observed an increase in the relative abundance of Lactococcus and Rikenella and a decrease in Holdemania, Ruminococcus_gauvreauii_group and Massilia in the NaDF group. Lactococcus are lactic acid-producing bacteria (LAB) that produce bacteriocins. Lactococcus has the “generally recognized as safe” (GRAS) status by the Food and Drug Administration (FDA), and has been widely used in livestock and poultry farming (71, 72). Rikenella is thought to be an anaerobic bacterium that capable of producing SCFAs, but its beneficial potential has not been reported in the literature (73). The increased relative abundance of beneficial bacteria caused by NaDF probably contributes to resistance to Salmonella in chicks.

The levels of the five major SCFAs in the duodenum and cecum did not significantly change with 1 g/kg NaDF supplementation in broilers’ diet. SCFAs are major end-products of complex carbohydrates fermented by anaerobic gut bacteria (74). Although the abundances of certain genera of bacteria in the intestine were altered, NaDF did not significantly affect the diversity of intestinal microbes, which may explain the unchanged levels of SCFAs. Therefore, we suggest that the acidification of the GIT and increased growth-related hormones are the main reasons for the growth-promoting effects of 1 g/kg NaDF supplemented to the chicken diet.

A stable environment in the intestine is crucial for a healthy host. Pathogenic microorganisms can be present in the gut for lasting periods at low concentrations, but without any negative effect on the host. However, some of the microbiota, despite their beneficial effects, can turn detrimental by producing toxic metabolites when the *in vivo* environment is unfavorable for their survival (75). Therefore, safe and sustainable feed additives need to have minimal impacts on the overall GIT microbiota composition (7). Inappropriate amounts of acid additives can impact the abundance of beneficial bacteria, with one significant challenge being their potential detrimental effect on LAB (17). For example, supplementation of 6 g/kg FA in the diet of piglets reduced the abundance of Lactobacilli in the intestine (76). Our previous study found a decrease in the relative abundance of Lactobacillus and Lactococcus in the duodenum of chicken upon the addition of 5 g/kg KDF to the feed (25). In the pre-experiment, we found that supplementation with 5 g/kg NaDF in broilers diet decreased the ADG and ADFI at the whole experimental period (1-42d) (data not shown). Based on these results and considering the common effective and safe dose of acidifiers already used in poultry farming, supplementation with 1 g/kg NaDF was chosen as the experimental dose in this study. We found that the addition of 1 g/kg NaDF to the feed did not affect the relative abundance of LAB, which is probably due to a reduction in supplementation amount. The acid tolerance evolution of pathogenic microorganisms is also a challenge of the application of acidifiers. Many intestinal pathogens have developed tolerance to acidic environments as a survival strategy. For example, the pathogenic *E. coli* and Salmonella show enhanced resistance to extreme acidic conditions after exposure to SCFAs (77, 78). Salmonella is capable of producing formate which serves as a diffusible signal to induce bacterial invasion of Hep-2 epithelial cells (79). *Campylobacter* can utilize FA as a substrate for respiratory energy metabolism (80, 81). We found that supplementation with 1 g/kg NaDF in broiler feed increased the relative abundance of some beneficial bacteria and decreased the abundance of certain potential pathogenic bacteria, while generally maintaining the stability of the diversity of intestinal commensal microbiota. Thus, the amount of NaDF used in this study can be considered both safe and effective in promoting the health of chickens.

## 5 Conclusion

In conclusion, as a novel formate acidifier, NaDF supplemented at 1 g/kg in diet for broilers lowered the pH values in the GIT, improved growth performance characterized by increased BW, decreased FCR and improved intestinal morphology. These effects are likely to result from increased ghrelin and GH secretions caused by NaDF. NaDF also modulated the abundances of certain intestinal bacteria without significantly changing the microbiota diversity and SCFAs levels, which would be beneficial for maintaining gut homeostasis during the sustainable use of NaDF. Additionally, NaDF effectively protected chickens from Salmonella infections evidenced by reduced bacterial loads in tissues and mitigated pathological changes after bacterial infection. This study provided evidence for the clinical application of NaDF in poultry production.

## Data availability statement

The datasets presented in this study can be found in online repositories. The names of the repository/repositories and accession number(s) can be found at: https://www.ncbi.nlm.nih.gov/, SRP472608.

## Ethics statement

The animal study was approved by Animal Experiment Ethics Committee of Huazhong Agricultural University (No: HZAUCH-2023-0018 and HZAUCH-2023-0019). The study was conducted in accordance with the local legislation and institutional requirements.

## Author contributions

YS: Conceptualization, Data curation, Formal analysis, Investigation, Methodology, Software, Writing – original draft. XZ: Formal analysis, Investigation, Methodology, Writing – original draft. WH: Formal analysis, Investigation, Methodology, Writing – original draft. WL: Formal analysis, Writing – original draft. JH: Formal analysis, Writing – original draft. YC: Formal analysis, Writing – original draft. HL: Formal analysis, Funding acquisition, Writing – review & editing. XC: Formal analysis, Funding acquisition, Writing – review & editing. QH: Formal analysis, Writing – review & editing. RZ: Formal analysis, Writing – review & editing. LL: Formal analysis, Funding acquisition, Supervision, Writing – original draft, Writing – review & editing.

## Footnotes

^1^Silva v138, http://www.arb-silva.de

^2^ https://cloud.majorbio.com

^3^ http://huttenhower.sph.harvard.edu/galaxy
