# Supplementary material for: Dietary supplementation with a novel acidifier sodium diformate improves growth performance by increasing growth-related hormones levels and prevents Salmonella enterica serovar Pullorum infection in chickens
Source: Front Vet Sci. 2024 Jul 17;11:1433514. doi: 10.3389/fvets.2024.1433514 (PMC11295659; doi:10.3389/fvets.2024.1433514)
Supplement: Supplementary file 1 [file Data_Sheet_1.docx]

Supplementary Material

# Supplementary Figure


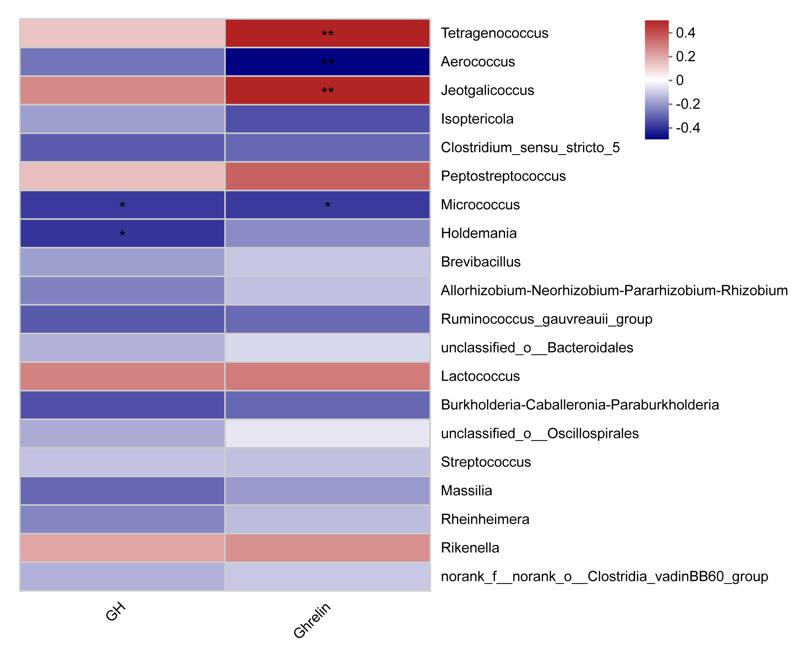


Figure S1 Spearman correlation heatmap. ^*^*P* < 0.05, ^**^ *P* < 0.01.

# Supplementary Tables

Table S1. Acidification capacity of NaDF.

| Volume of NaOH solution (mL) | NaDF | KDF |
| --- | --- | --- |
| (Before the sudden change in pH) | | |
| 0 | 3.45±0.02 | 3.54±0.01 |
| 12 | 3.76±0.02 | 3.85±0.01 |
| 24 | 4.16±0.02 | 4.37±0.02 |
| 30 | 4.53±0.05 ^a^ | 5.15±0.1^b^ |
| (After the sudden change in pH) | | |
| 35.82±0.69 ^a^ (NaDF) | 8.47±0.35 |  |
| 31.67±0.31 ^b^ (KDF) |  | 8.42±0.33 |

^a, b^ Means significant differences between NaDF and KDF groups

Table S2. Buffering capacity of NaDF.

| Groups | Masses (g) | | | | | | | | |
| --- | --- | --- | --- | --- | --- | --- | --- | --- | --- |
|  | 0.02 | 0.2 | 1 | 2 | 5 | 10 | 20 | 50 | 100 |
| NaDF | 3.80±0.01 | 3.63±0.01 | 3.56±0.01 | 3.53±0.01 | 3.49±0.01 | 3.46±0.01 | 3.44±0.01 | 3.44±0.01 | 3.50±0.01 |
| KDF | 3.85±0.01 | 3.67±0.01 | 3.60±0.01 | 3.58±0.01 | 3.56±0.01 | 3.55±0.01 | 3.57±0.01 | 3.67±0.02 | 3.85±0.01 |

Table S3. Weekly growth performance of broilers fed with NaDF.

| Items | Con | NaDF | *P* value |
| --- | --- | --- | --- |
| *BW, g* | | | |
| d 1 | 40.48±0.01 | 40.48±0.00 | 0.3343 |
| d 7 | 179.62±3.53^a^ | 183.09±2.98^b^ | ^*^0.0517 |
| d 14 | 467.46±12.43 | 473.71±11.55 | 0.3144 |
| d 21 | 798.55±25.23 | 813.14±23.45 | 0.2509 |
| d 28 | 1372.74±37.34^a^ | 1414.94±32.41^b^ | ^*^0.0300 |
| d 38 | 2293.8±45.69^a^ | 2342.98±45.42^b^ | ^*^0.0487 |
| *ADG, g/d per bird* | | | |
| d 1-7 | 19.85±0.53 | 20.31±0.44 | 0.0756 |
| d 8-14 | 41.04±1.65 | 41.43±1.52 | 0.6368 |
| d 15-21 | 47.17±2.43 | 48.16±3.27 | 0.5015 |
| d 22-28 | 81.06±30^a^ | 85.25±2.61^b^ | ^**^0.0100 |
| d 29-38 | 89.68±3.80 | 91.18±4.13 | 0.4630 |
| d 22-38 | 101.16±2.86 | 103.22±2.87 | 0.1731 |
| d 1-14 | 30.42±0.90 | 30.85±0.87 | 0.3527 |
| d 1-21 | 35.98±1.26 | 36.6±1.26 | 0.3430 |
| d 1-28 | 47.11±1.55^a^ | 48.63±1.27^b^ | ^*^0.0500 |
| d 1-38 | 58.12±1.89 | 59.67±1.59 | 0.0976 |
| *ADFI, g/d per bird* | | | |
| d 1-7 | 21.37±0.38 | 21.39±0.33 | 0.9400 |
| d 8-14 | 49.86±1.29 | 50.71±1.32 | 0.2135 |
| d 15-21 | 73.52±3.30 | 74.84±3.18 | 0.4314 |
| d 22-28 | 117.38±2.78^a^ | 120.26±2.63^b^ | ^*^0.0514 |
| d 29-38 | 156.65±5.7 | 156.68±3.05 | 0.9889 |
| d 22-38 | 140.37±4.01 | 141.6±2.50 | 0.1069 |
| d 1-14 | 35.59±0.62 | 36.02±0.70 | 0.2094 |
| d 1-21 | 48.18±1.39 | 48.92±1.29 | 0.2938 |
| d 1-28 | 65.27±1.63 | 66.57±1.59 | 0.1294 |
| d 1-38 | 88.91±2.78 | 89.94±2.04 | 0.4121 |
| *F/G (g/g)* | | | |
| d 1-7 | 1.08±0.01^a^ | 1.05±0.02^b^ | ^**^0.0068 |
| d 8-14 | 1.22±0.02 | 1.22±0.02 | 0.4928 |
| d 15-21 | 1.56±0.02 | 1.56±0.05 | 0.8208 |
| d 22-28 | 1.45±0.03^a^ | 1.41±0.02^b^ | ^**^0.0090 |
| d 29-38 | 1.75±0.02 | 1.72±0.05 | 0.2161 |
| d 22-38 | 1.39±0.01 | 1.37±0.02 | 0.0792 |
| d 1-14 | 1.17±0.02 | 1.16±0.01 | 0.8814 |
| d 1-21 | 1.34±0.02 | 1.34±0.02 | 0.7884 |
| d 1-28 | 1.39±0.02 | 1.37±0.01 | 0.0594 |
| d 1-38 | 1.53±0.01^a^ | 1.51±0.01^b^ | ^**^0.0011 |

Different superscripts in the same row showed significant difference. Con group (basal diets); NaDF group (NaDF in diet). Abbreviations: BW, Body Weight; ADG, average daily gain; ADFI, average daily feed intake; F/G, feed conversion ratio. ^*^*P* < 0.05, ^**^ *P* < 0.01.

Table S4 Concentrations of SCFAs in the duodenum and cecum of broilers (μg/g).

| Items | Acetic acid | Propanoic acid | Butanoic acid | Valeric acid | Hexanoic acid |
| --- | --- | --- | --- | --- | --- |
| Duodenum |  |  |  |  |  |
| Con | 50.53±21.10 | 1.94±1.38 | 1.35±1.13 | 0.50±0.22 | 0.82±0.25 |
| NaDF | 63.01±84.40 | 2.09±0.53 | 0.85±0.17 | 0.34±0.07 | 0.80±0.21 |
| *P* value | 0.6911 | 0.7745 | 0.2402 | 0.0763 | 0.8388 |
| Cecum |  |  |  |  |  |
| Con | 7362.83±1732.19 | 1614.55±465.49 | 2826.59±609.6 | 353.55±118.78 | 11.36±4.28 |
| NaDF | 6580.69±757.45 | 1639.12±466.58 | 2417.14±701.23 | 271.33±86.58 | 7.97±2.83 |
| *P* value | 0.2615 | 0.9175 | 0.2331 | 0.1359 | 0.0827 |

Con group (basal diets); NaDF (NaDF in diet).

Table S5. MIC of NaDF against common pathogenic bacteria on the farm.

| Categories | Genus | Species/strains | MIC (mg·mL^-1^) |
| --- | --- | --- | --- |
| gram-negative bacteria | Salmonella | *S. typhimurium* ATCC 14028 | 3.1250 |
|  |  | *S. typhimurium* CVCC 212197 | 3.1250 |
|  |  | *S. 1typhimurium* CVCC 542 | 3.1250 |
|  |  | *S.* Pullorum C79-3 | 3.1250 |
|  | Escherichia | *E. coli* ATCC25922 | 3.1250 |
|  |  | *E. coli* PCN033 | 3.1250 |
|  |  | *E. coli* 72 | 3.1250 |
|  |  | *E. coli* 7 | 3.1250 |
|  | Pasteurella | *P. multocida* 9261 | 3.1250 |
|  |  | *P. multocida* HB03 | 1.5625 |
|  | Actinobacillus | *A. pleuropneumoniae* 4074 | 3.1250 |
| gram-positive bacteria | Staphylococcus | *S. aureus* ATCC212193 | 6.2500 |
|  |  | *S. aureus* 1213M4A | 3.1250 |
|  | Streptococcus | *S. suis* SC19 | 1.5625 |
|  | Clostridium | *C. perfringens ML2* | 3.1250 |

Abbreviation: *MIC*, minimum inhibitory concentration.
